# Performance comparison of two commercial human whole-exome capture systems on formalin-fixed paraffin-embedded lung adenocarcinoma samples

**DOI:** 10.1186/s12885-016-2720-4

**Published:** 2016-08-30

**Authors:** Silvia Bonfiglio, Irene Vanni, Valeria Rossella, Anna Truini, Dejan Lazarevic, Maria Giovanna Dal Bello, Angela Alama, Marco Mora, Erika Rijavec, Carlo Genova, Davide Cittaro, Francesco Grossi, Simona Coco

**Affiliations:** 1 Centre for Translational Genomics and Bioinformatics, San Raffaele Scientific Institute, Via Olgettina 58, Milan, 20132 Italy; 2 Lung Cancer Unit, IRCCS AOU San Martino - IST National Cancer Research Institute, L.go R. Benzi 10, Genoa, 16132 Italy; 3 Department of Internal Medicine and Medical Specialties (DIMI), University of Genoa, Italy, IRCCS AOU San Martino - IST National Cancer Research Institute, L.go R. Benzi 10, Genoa, 16132 Italy; 4 Department of Pathology, IRCCS AOU San Martino - IST National Cancer Research Institute, L.go R. Benzi 10, Genoa, 16132 Italy

**Keywords:** Exome sequencing, FFPE, Quality control, Solution-based capture, Cancer-related genes, Lung adenocarcinoma

## Abstract

**Background:**

Next Generation Sequencing (NGS) has become a valuable tool for molecular landscape characterization of cancer genomes, leading to a better understanding of tumor onset and progression, and opening new avenues in translational oncology. Formalin-fixed paraffin-embedded (FFPE) tissue is the method of choice for storage of clinical samples, however low quality of FFPE genomic DNA (gDNA) can limit its use for downstream applications.

**Methods:**

To investigate the FFPE specimen suitability for NGS analysis and to establish the performance of two solution-based exome capture technologies, we compared the whole-exome sequencing (WES) data of gDNA extracted from 5 fresh frozen (FF) and 5 matched FFPE lung adenocarcinoma tissues using: SeqCap EZ Human Exome *v*.3.0 (Roche NimbleGen) and SureSelect XT Human All Exon *v*.5 (Agilent Technologies).

**Results:**

Sequencing metrics on Illumina HiSeq were optimal for both exome systems and comparable among FFPE and FF samples, with a slight increase of PCR duplicates in FFPE, mainly in Roche NimbleGen libraries. Comparison of single nucleotide variants (SNVs) between FFPE-FF pairs reached overlapping values >90 % in both systems. Both WES showed high concordance with target re-sequencing data by Ion PGM™ in 22 lung-cancer genes, regardless the source of samples. Exon coverage of 623 cancer-related genes revealed high coverage efficiency of both kits, proposing WES as a valid alternative to target re-sequencing.

**Conclusions:**

High-quality and reliable data can be successfully obtained from WES of FFPE samples starting from a relatively low amount of input gDNA, suggesting the inclusion of NGS-based tests into clinical contest. In conclusion, our analysis suggests that the WES approach could be extended to a translational research context as well as to the clinic (e.g. to study rare malignancies), where the simultaneous analysis of the whole coding region of the genome may help in the detection of cancer-linked variants.

**Electronic supplementary material:**

The online version of this article (doi:10.1186/s12885-016-2720-4) contains supplementary material, which is available to authorized users.

## Background

The advent of Next Generation Sequencing (NGS) technology has revolutionized the knowledge of cancer genomics becoming a valuable tool to characterize the molecular landscape of cancer genomes in different tumor types, including lung cancer [1–3]. NGS allows to comprehensively identifying genetic variants associated with individual cancer leading to a better understanding of tumor onset and progression, opening new avenues in the field of translational oncology [4–6].

Whole Exome Sequencing (WES), which targets a large fraction of the protein coding region of the genome, is a widely used sequencing strategy. Indeed, it is a cost-effective approach compared to the prohibitively expensive whole genome sequencing and a valid alternative to gene panels [7–10]. However, WES is still relatively expensive and it requires bioinformatic expertise for data analysis; moreover, one of the major challenges is represented by the quality and integrity of nucleic acid extracted from available tumor tissues. The best source of samples is fresh frozen (FF) sections, which results in high quality DNA, although handling and storage often limit the possibility to perform molecular analyses including NGS. To date, formalin-fixed paraffin-embedded (FFPE) preservation is the method of choice for the archival storage of clinical samples in pathology archives worldwide. Although the FFPE tumor tissue might be an excellent resource for retrospective and prospective molecular genetic investigations, the low quality of resulting DNA remains one of the major challenges. The difficulty of extraction due to paraffin and protein-DNA interactions, together with the adverse effect of formalin fixatives, could result in chemical modification and fragmentation of FFPE-derived DNA, limiting its use for downstream applications [11–13]. In 2009, Schweiger and colleagues for the first time successfully demonstrated the possibility to obtain copy-number alterations and mutation data using long-term storage FFPE samples without any significant drawback when compared to matched FF samples [14].

During the five past years, noteworthy efforts have been made to establish the performance of different exome capture systems and help define the most appropriate capture system for each specific application [15–21]. In addition, several groups evaluated the FFPE-derived gDNA suitability in WES applications [22–28] (Table 1). At present only two systematic comparisons of different exome capture technologies performance on FF and matched FFPE tissues have been published [27, 28], however the comparison analyses were carried out on different sets of samples, providing unclear results (Table 1).

**Table 1:** Overview of the most relevant WES comparison studies between FF and matched FFPE tissue samples

Study	Number/Sample types	Tissue type	Exome capture kit
Holley et al. [22]	1 matched FF/FFPE	pancreatic ductal adenocarcinoma	Agilent SureSelect All Exon Plus
Van Allen et al. [23]	11 matched FF/FFPE	lung adenocarcinoma + lung normal tissue	Agilent SureSelect Human All Exon *v.2*
Hedegaard et al. [24]	19 matched FF/FFPE	colorectal carcinoma + 13 matching normal FF colon samples	Illumina TruSeq Exome Enrichment
Munchel et al. [25]	13 matched FF/FFPE	9 ovarian carcinomas, 2 breast tumor/normal pairs, 2 colon tumor/normal pairs	Illumina TruSeq Exome Enrichment
Astolfi et al. [26]	4 matched FF/FFPE	gastrointestinal stromal tumors + normal samples (peripheral blood)	Illumina Nextera Rapid Capture Exome Enrichment
De Paoli-Iseppi et al. [27]	10 matched FF/FFPE	melanoma	Illumina TruSeq Exome (10 FF)
			Illumina Nextera Rapid Capture Expanded Exome (7 FFPE)
			Roche NimbleGen SeqCap EZ Exome +UTR (4 FFPE)
Oh et al. [28]	4 matched FF/FFPE	cancer type not defined + matched blood or normal frozen sample	NimbleGen exome 2.1 M array (pair 1 and 4);
			Agilent SureSelect All Human exon *v.5* (pair 2 and 3).

Currently, the most used exome enrichment platforms are characterized by the solution-based capture technology and Roche NimbleGen and Agilent SureSelect are two out of the four major commercially available platforms [17, 21].

Here we present a comprehensive comparison of the Roche NimbleGen SeqCap EZ Exome (*v*.3.0; 64 Mb) and Agilent SureSelect XT (*v*.5; 50 Mb) (Table 2), on genomic DNA (gDNA) extracted from FF and matched FFPE tissue belonging to five lung adenocarcinoma (ADC) patients.

**Table 2:** Comparison between Agilent SureSelect XT *v*.5 and Roche NimbleGen *v*3.0 exome capture systems

	Agilent SureSelect XT *v.*5	Roche NimbleGen *v.*3.0
Probe type	biotinylated cRNA	biotinylated DNA
Probe length range (bp)	114-126	55-105
Number of probes	~655,872	>2,100,000
Probe design	non-overlapping (adjacent)	overlapping
Total target length (Mb)	50	64

A gDNA integrity quality control step was also included to determine the suitability of FFPE tumor specimens for WES analysis on Illumina HiSeq platform. Furthermore, we compared WES data with PCR-based target re-sequencing, evaluating the variant calling concordance of 90 amplicons within 22 lung cancer-related genes included in the Ion AmpliSeq Colon and Lung Cancer Panel *v.*1 (Thermo Fisher Scientific). Finally, we also assessed the uniformity of coverage reached by the two exome enrichment platforms in 623 cancer-related genes.

## Methods

### Clinical samples

Tissue samples were obtained from five patients diagnosed with histologically confirmed lung ADC who underwent surgery (2 IB, 2 IIB and 1 patient IV stage of disease). For each patient, FF and matched FFPE samples were collected from the Biological Resource Center (CRB) and from diagnostic archive of IRCCS A.O.U. San Martino – IST (Genova, Italy), respectively. Each tumor sample was evaluated by pathologist prior to analysis and all specimens reported at least 50 % of tumor cells content.

### DNA extraction and quality control

gDNA from FF and matched FFPE tissues was extracted by QIAamp® DNA Mini Kit and GeneRead DNA FFPE Kit (Qiagen, Hilden, Germany), respectively. Quantity and purity of gDNA were assessed by Qubit® 2.0 Fluorometer (Invitrogen, Carlsbad, CA, USA) and NanoDrop ND-1000 (Thermo Scientific, Wilmington, DE, USA). Fragmentation status was evaluated by the Agilent 2200 TapeStation system using the Genomic DNA ScreenTape assay (Agilent Technologies, Santa Clara, CA, USA) able to produce a DNA Integrity Number (DIN). An additional quality control (QC) step to assess FFPE DNA integrity was performed using a multiplex Polymerase Chain Reaction (PCR) approach [29]. Briefly, 30 ng of gDNA were amplified using three different-size set of primers of Glyceraldehyde-3-Phosphate Dehydrogenase (*GAPDH*) gene (200-300-400 base pair), and the concentration of PCR products was determined by Agilent 2100 Bioanalyzer instrument (Agilent Technologies). Then, to estimate FFPE gDNA fragmentation, we evaluated an Average Yield Ratio (AYR) value, calculated by yield ratio of each amplicon compared with a reference DNA (Promega Madison, WI, USA).

### WES library preparation and hybridization capture

A total of 300 ng of each gDNA sample based on Qubit quantification were mechanically fragmented on a E220 focused ultrasonicator Covaris (Covaris, Woburn, MA, USA). Two hundred ng of sheared gDNA were used to perform end repair, A-tailing and adapter ligation with either Agilent SureSelect XT (Agilent Technologies) or KAPA library preparation kits (Kapa Biosystems Inc. Wilmington, MA, USA), following the manufacturer instructions. Subsequently, the libraries were captured using either Agilent SureSelect Human All Exon *v.*5 (Agilent Technologies) or SeqCap EZ Human Exome Library *v.*3.0 Roche NimbleGen (Roche, Basel, Switzerland) probes respectively, and finally amplified.

### Illumina sequencing

After QC and quantification by Agilent 2100 Bioanalyzer (Agilent Technologies) and Qubit® 2.0 Fluorometer (Invitrogen), the libraries were sequenced on an Illumina HiSeq 2500 platform (Illumina Inc, San Diego, CA, USA) High Output mode, 2×100 cycles, with TruSeq SBS v3 chemistry. For each library preparation type, 10 samples were loaded in a single lane of a flow-cell v3.

### WES data analysis and statistical analysis

After sequencing, basecall files conversion and demultiplexing were performed with bcl2fastq software (Illumina). The resulting fastq data were aligned to the human reference genome (hg19) by Burrows-Wheeler Aligner Maximal Exact Match (BWA-MEM) aligner [30]. We assessed duplicated reads with Picard MarkDuplicates; Picard HsMetrics [31] and Samtools [32] were used to determine WES metrics. Reads realignment and base recalibration were performed with the Genome Analysis Toolkit (GATK) tools InDelRealigner and BaseRecalibrator. Recalibrated Binary Alignment/Map (BAM) files were used to perform variant calling with the GATK-UnifiedGenotyper [33]. Two tails paired t and ANOVA tests were performed by Microsoft Excel.

### Selection of genes implicated in cancer

In order to select the most relevant cancer-related genes, we focused on 5 different companies releasing commercial re-sequencing panels. The selected 21 panels are the following: Ion AmpliSeq™ Cancer Hotspot Panel *v*.2, Ion AmpliSeq™ Colon and Lung Research Panel *v.2,* Ion AmpliSeq™ Comprehensive Cancer Panel, Ion AmpliSeq™ Cancer Panel Primer Pool (Thermo Fisher Scientific); TruSeq™ Amplicon Cancer Panel, TruSight™ Tumor Panel (llumina Inc); Human Breast Cancer Panel, Human Colorectal Cancer Panel, Human Liver Cancer Panel, Human Lung Cancer Panel, Human Ovarian Cancer Panel, Human Prostate Cancer Panel, Human Gastric Cancer Panel, Human Cancer Predisposition Panel, Human Clinically Relevant Tumor Panel, Human Tumor Actionable Mutations Panel, Human Comprehensive Cancer Panel (Qiagen), Somatic 1 MASTR *v.2*, Somatic 2 MASTR Plus (Multiplicom, Niel, Belgium); Clear Seq Comprehensive Cancer and Clear Seq Cancer (Agilent Technologies).

### Coverage analysis of cancer genes

A total of 623 cancer-related genes was used to analyze the coverage performance of WES enrichment systems by the DiagnoseTargets tool from GATK. We set the tool parameters in order to identify a ‘critical’ exon interval in a single library when the average depth of coverage was less than 10× for at least 20 % of the exon interval length. Finally, for each kit, all the intervals with insufficient median depth across all FF and FFPE libraries were considered ‘critical’.

The region coordinates (RefSeq coding exons) were downloaded from UCSC Table Browser [34]. BEDTools [35] was used to collapse coordinates to unique locations in order to avoid overlap.

### Target resequencing for WES validation

For targeted NGS analysis, the libraries were constructed using the Ion AmpliSeq Colon and Lung Cancer Panel *v.*1 (Thermo Fisher Scientific) which amplifies 90 amplicons in hotspot regions of 22 Colon and Lung cancer-related genes (*AKT1, ALK, BRAF, CTNNB1, DDR2, EGFR, ERBB2, ERBB4, FBXW7, FGFR1, FGFR2, FGFR3, KRAS, MAP2K1, MET, NOTCH1*, *NRAS*, *PIK3CA*, *PTEN*, *SMAD4*, *STK11,* and *TP53*). gDNA extracted from FFPE and FF samples (20 ng and 10 ng, respectively) were amplified using the Ion AmpliSeq™ Library Kit 2.0 (Thermo Fisher Scientific) according to the manufacturer's instructions. After libraries quantification and QC, performed by the 2200 TapeStation Instrument (High Sensitivity Assay) and Qubit® 2.0 Fluorometer, each library was diluted to 100pM, amplified through emulsion PCR using the OneTouch™ Instrument (Thermo Fisher Scientific) and enriched by the OneTouch™ ES Instrument (Thermo Fisher Scientific) using the Ion PGM Template OT2 200 KIT following manufacturer’s instructions. The targeted resequencing was carried out on the Ion Personal Genome Machine (PGM) sequencer (Ion Torrent™) using the Ion PGM 200 Sequencing Kit (Thermo Fisher Scientific) loading barcoded libraries into 316*v.*2 chip. Sequencing was performed using 500 flow runs generating approximately 200 bp reads. The PGM sequencing data analysis was performed by the Ion Torrent Software Suite *v.*4.2 (Thermo Fisher Scientific) using the plugin Variant Caller (VC) *v.*4.2-r88446. The called variants were annotated by the Ion Reporter software *v.*4.2 and verified using the Integrative Genomics Viewer (IGV) software.

## Results

### Quality control

gDNA was extracted from 5 FF and matched FFPE samples. A QC step was performed for each sample (Additional file 1: Figure S1). FFPE gDNA fragmentation status was evaluated using a multiplex PCR and an automated gel-based electrophoresis system (2200 TapeStation Instrument; Agilent Technologies) reporting variable degradation status: the multiplex PCR revealed an AYR ranging from 0.5–0.7, whereas the TapeStation reported a DIN which ranged from 3.5–4.3. The AYR values highly correlated with DIN data, although the two systems reported different scales of measurement.

### WES standard metrics comparison

**Fig. 1:**
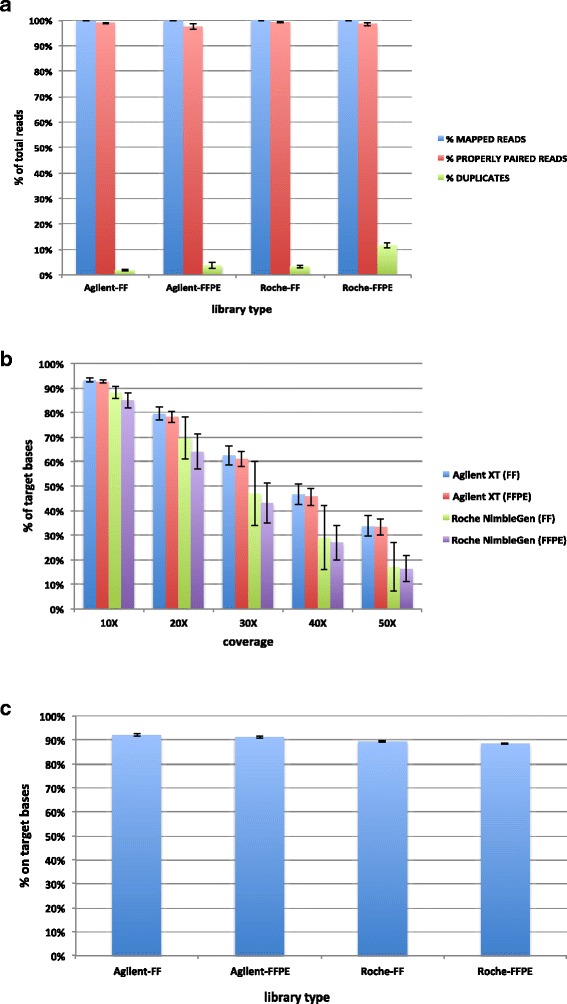
WES metrics comparison. Mean percentage ± SD (*n* = 5) of mapped, properly paired and duplicated reads obtained for each exome capture technology in both FF and FFPE libraries (**a**). Mean percentage ± SD (*n* = 5) of target bases achieving a certain coverage value or higher for each library type suggests that Roche kit tends to accumulate reads in low coverage regions (**b**). Mean percentage ± SD (*n* = 5) of on target bases for each library type. On target bases are referred to the number of aligned bases that map either on or near a bait within a 100 bp interval (**c**)

WES was performed on all samples (5 FF and matching FFPE), comparing two commercially available exome capture systems: Roche NimbleGen SeqCap EZ Human Exome Library *v.*3.0 (64 Mb) and Agilent SureSelect Human All Exon *v.*5 (50 Mb). The standard WES metrics, computed for each library, are summarized in Additional file 2: Table S1. No major differences were found between FF and FFPE libraries, and both exome capture systems showed a similar sequencing performance (Fig. 1). The percentage of reads mapping to the reference genome was higher than 99 % for both sample types, irrespective of the kit used (Fig. 1a, Additional file 2: Table S1). Also the mean percentage of properly paired reads was comparable, showing a value of 98.9 % (range 98.3-99.1) and 97.4 % (range 95.3-98.1) in FF and FFPE Agilent libraries respectively, and 99.1 % (range 98.7-99.3) and 98.5 % (range 97.6-98.9) in FF and FFPE Roche NimbleGen libraries respectively (Fig. 1a, Additional file 2: Table S1). A slightly higher percentage of duplicated reads was obtained in FFPE compared with FF libraries for both exome capture kits. However, overall Roche NimbleGen technology achieved a higher level of duplicated reads (FF mean = 3.3 %; FFPE mean = 11.5 %) as compared to Agilent SureSelect kit (FF mean = 1.8 %; FFPE mean = 3.6 %) (Fig. 1a, Additional file 2: Table S1). The percentage of duplicated reads was higher in FFPE compared with FF libraries for both exome capture kits (*p* = 0.01 for Agilent SureSelect, *p* = 1.6*10^-4^ for Roche NimbleGen, two tails paired *t* test). Overall, Roche NimbleGen technology showed a higher level of duplicated reads than Agilent SureSelect for both FF (*p* = 0.01, two tails paired *t* test) and FFPE samples (*p* = 1.6*10^-4^, two tails paired *t* test) (Fig. 1a, Additional file 2: Table S1).

Despite the higher number of PCR-duplicates in FFPE samples, the mean target coverage, estimated without duplicated reads, showed similar results for FF and FFPE samples. Specifically, the mean values achieved in Agilent libraries were 44.2× (range 40.7-48.4) and 44.5× (range 41.0-47.8) for FF and FFPE libraries respectively, whereas for Roche NimbleGen kit the mean values were 33.8× (range 27.7-44.9) and 31.9× (range 26.5-37.4) for FF and FFPE libraries, respectively (Additional file 2: Table S1). Overall, the total number of reads was generally lower for Agilent libraries. The higher mean target coverage achieved in Agilent libraries was not surprising, as the kit intended target region covers 50 Mb of the genome, compared to the 64 Mb target region covered by Roche NimbleGen kit. However, even taking into account the difference in the target region length, the mean target coverage achieves a better performance in Agilent kit with respect to the number of reads per sample. Moreover, when we considered the percentage of target bases achieving at least a certain coverage threshold, the Agilent SureSelect kit showed a better performance. In particular, on average, more than 90 % of intended target region exhibited at least 10× coverage in both FF and FFPE Agilent libraries compared with 88 % (FF) and 85 % (FFPE) of target which had at least 10× coverage in Roche NimbleGen libraries (Fig. 1b). Finally, the percentage values of bases on target are higher in FF than FFPE libraries in both exome platforms (*p* = 0.03 for Agilent SureSelect, *p* = 0.04 for Roche NimbleGen, two tails paired *t* test), and show a better performance of Agilent SureSelect kit over the Roche NimbleGen kit for both FF (*p* = 1.1*10^-4^, two tails paired *t* test) and FFPE samples (*p* = 1.5*10^-4^, two tails paired *t* test) (Fig. 1c, Additional file 2: Table S1).

### Variant detection and genotype comparison between FF and FFPE samples

**Fig. 2:**
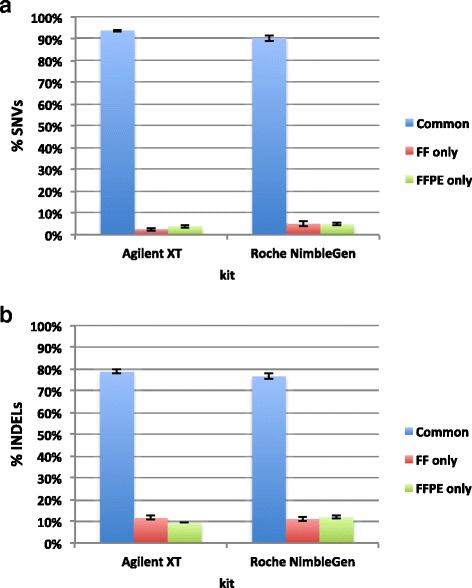
Variant calling comparison between FF and FFPE samples. The mean ± SD, computed across five matched FF-FFPE pairs, of the percentage of SNVs (**a**) and InDels (**b**) common to both sample types (blue) and unique to either FF (red) or FFPE (green) samples is reported for both capture systems. They both show on average ≥ 90 % of shared SNVs, and < 80 % of common InDels between FF and FFPE samples

**Fig. 3:**
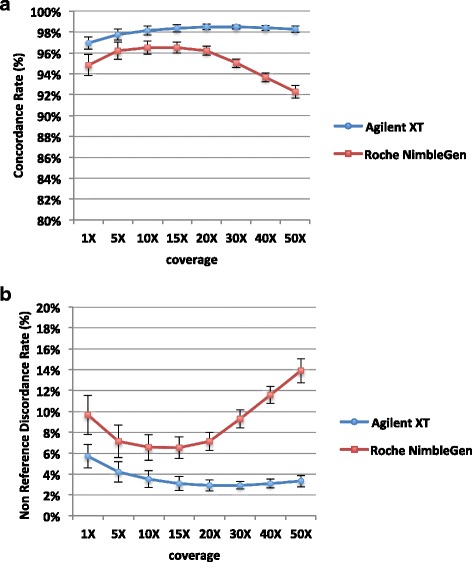
Genotype concordance (CR) and non-reference discordance (NRDR) rates between matched FF-FFPE pairs computed at increasing coverage thresholds. The mean ± SD across five matched FF-FFPE pairs of the CR % (**a**) or of the NRDR % (**b**) is reported at each coverage threshold for both Agilent and Roche kit

To assess the suitability of FFPE samples for WES analysis, we determined the total number of SNVs and Insertion/Deletions (InDels) in all FF-FFPE pairs. Then, we determined the number of variants in common between both sample types and unique to either FF or FFPE sample (Fig. 2, Additional file 2: Table S2). On average, both capture system kits showed a percentage of shared SNVs higher than 90 % (Fig.2a, Additional file 2: Table S2); whereas the average percentage of common InDels within each pair was lower than 80 % (Fig.2b, Additional file 2: Table S2). This data might be probably due to the GATK variant caller, which requires higher coverage to accurately call InDels compared to SNVs, as suggested by Wong et al. [36]. Moreover, we determined the genotype concordance rate (CR) and non-reference discordance rate (NRDR) between each matched FF-FFPE pair at different coverage thresholds, for both exome capture systems. As shown in Additional file 2: Table S3a and in Fig. 3a, for Agilent SureSelect kit the average CR across all the five matched pairs was quite constant (≥97 %) across all coverage thresholds. Similarly, NRDR reported unvaried trend with a weak decrease from 6 % to 3 % at increasing coverage cut-offs (Additional file 2: Table S3b, Fig. 3b). For Roche NimbleGen kit, the average CR was lower than Agilent SureSelect kit (*p* = 1.42*10^-17^, ANOVA two-factor without replication), with a reduction from 95 % to 92 % at increasing coverage cut-offs (Additional file 2: Table S3a, Fig. 3a); similarly, the average NRDR values resulted worse in Roche NimbleGen libraries (*p* = 1.33*10^-18^, ANOVA two-factor without replication), with an increase at higher coverage cut-offs (Additional file 2: Table S3b, Fig. 3b).

In order to determine if FFPE samples were significantly enriched of FFPE artefacts (C > T and G > A substitutions), for both kits we computed CR and NRDR between each matched FF-FFPE pair at increasing coverage thresholds for each transition type (Additional file 2: Table S4). CR computed for either C > T or G > A substitutions was not significantly different (p-value <0.01) from the rate of the other transition types (A > G, T > C). The only exception was C > T compared to T > C in Agilent SureSelect kit at the highest coverage threshold (Additional file 2: Table S4a). Similarly, NRDR values computed for either C > T or G > A substitutions were not significantly different (p-value <0.01) from other transition types (A > G, T > C), although as coverage threshold increases (≥30×), in both kits the NRDR metric is able to spot significant differences due to cytosine deamination (Additional file 2: Table S4b). In Agilent SureSelect kit the NRDR values for C > T and G > A were twice the values of other transitions at 50× but still under 5 %.

### Variant detection and genotype comparison between exome capture systems

**Fig. 4:**
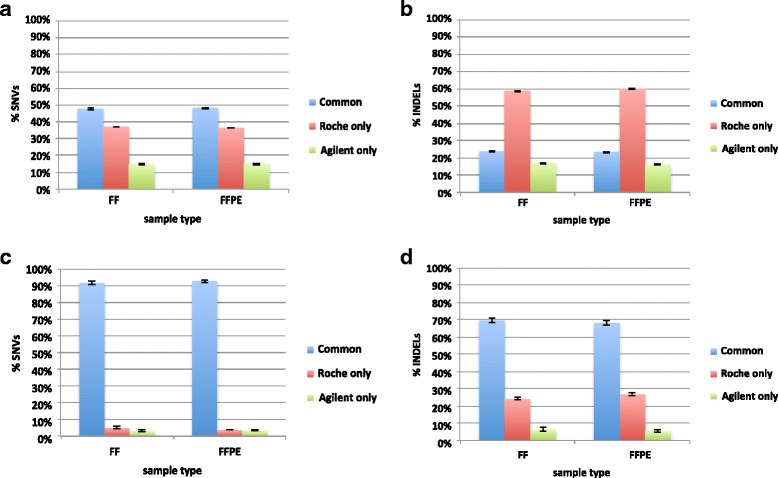
Variant calling comparison between Agilent SureSelect and Roche NimbleGen kit. Mean percentage ± SD of SNVs and InDels common to both library prep kits (blue), and private to either Roche (red) or Agilent (green) kit in both FF and FFPE samples. The average percentage of common SNVs (**a**) and InDels (**b**) was approximately 48 % (FF: 47.8 %; FFPE: 48.5 %) and 24 % (FF: 24 %; FFPE: 23.5 %) across the whole target region specific for each kit. The average percentage of common SNVs (**c**) and InDels (**d**) was approximately 92 % (FF: 91.9 %; FFPE: 93 %) and 69 % (FF: 69.7 %; FFPE: 69.1 %) across the 42 Mb target region shared between the two kits

We systematically compared the ability of the two exome capture systems to identify genomic variants. To this end, we determined the percentage of SNVs and InDels detected by both Agilent SureSelect and Roche NimbleGen kits across either their own target regions of 50 Mb and 64 Mb respectively (Fig. 4 a, b), or the common target region of 42 Mb (Fig. 4 c, d), for each FF and FFPE sample. When comparing the variant calling performance of the two kits across their whole specific target regions, the average percentage of common SNVs and InDels was approximately 48 % and 24 % respectively in both FF and FFPE samples (Fig. 4 a, b; Additional file 2: Table S5). This result was expected, since the two systems share almost half of the total enrichment space (42 Mb over a total of 72 Mb). When we considered this specific shared region for the comparison, the average percentages of common SNVs and InDels were found to be 92.4 % (FF: 91.9 %; FFPE: 93 %) and 68.9 % (FF: 69.7 %; FFPE: 68.1 %), respectively (Fig. 4 c, d, Additional file 2: Table S5). Furthermore, for each FF and FFPE sample, we computed CR and NRDR across the 42 Mb region shared between the two platforms (Additional file 2: Table S6). The average CR is ≥97 % and 98 % in FF and FFPE samples respectively, and it slightly decreases at coverage thresholds ≥ 40× (Additional file 2: Table S6a); similarly, NRDR is on average 5 % and 4 % in FF and FFPE samples respectively, increasing at coverage cut-offs ≥ 40× (Additional file 2: Table S6b).

### Variant detection comparison between WES and AmpliSeq Colon and Lung Cancer Panel

**Fig. 5:**
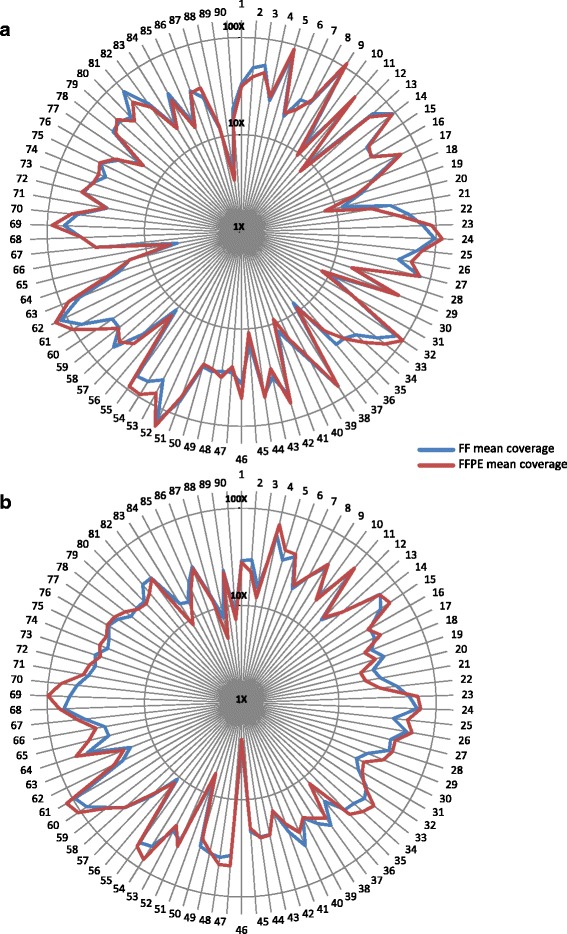
Coverage distribution across 90 PCR-capture amplicons between FF and FFPE samples. Coverage distribution across the 90 ‘AmpliSeq Colon and Lung Cancer Panel’ regions displays a similar trend between the FF (blue) and FFPE (red) libraries in both Agilent SureSelect (**a**) and Roche NimbleGen (**b**) libraries respectively, with a slightly better coverage in FFPE samples. Each amplicon is identified by a number as reported in Additional file 3: TableS7

**Fig. 6:**
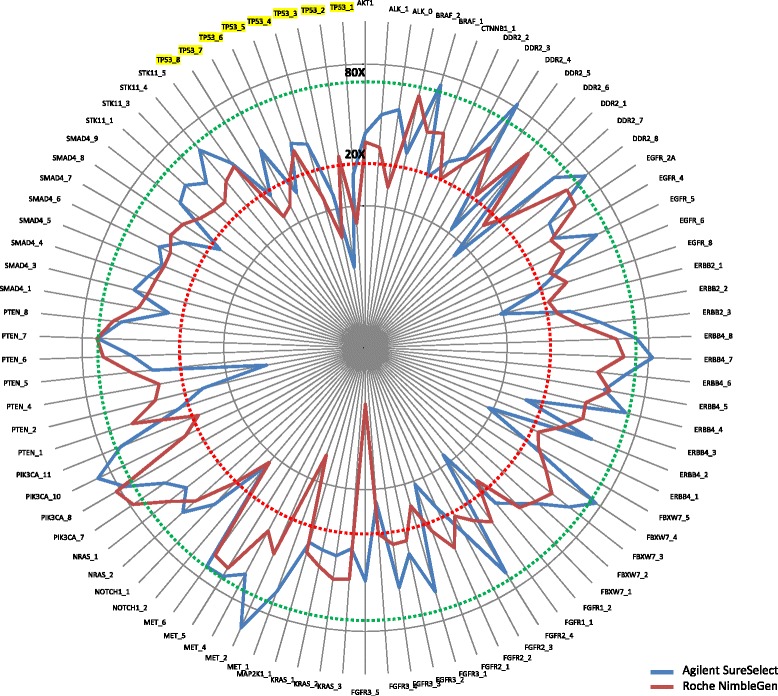
Comparison of coverage distribution across 90 PCR-capture amplicons of both WES systems. The comparison shows a lower uniformity across the amplicons in Agilent libraries, with a higher number of low read depth regions (20 amplicons with coverage <20× *vs* 14 of Roche) or very high coverage (10 amplicons with coverage >80× *vs* 2 of Roche). Both whole exome capture systems showed a poor coverage in *TP53* with 5/8 unsuccessfully covered amplicons (<20×) in each WES system. Coverage values were transformed in logarithmic scale

All samples included in the study were previously characterized using the “Ion AmpliSeq Colon and Lung Cancer Panel *v.*1” (Thermo Fisher Scientific) that screens targeted regions of 22 lung cancer-related genes, and sequenced by Ion Torrent PGM™ platform. In order to assess the concordance between WES and target PCR-based re-sequencing, we first examined the enrichment performance of the two WES kits. To do this we evaluated the mean coverage achieved by both capturing systems within the 90 PCR-captured regions contained in the 22 genes of interest (Additional file 3: Table S7). Considering the mean coverage across all the 90 regions, the Agilent SureSelect kit was found to have a higher mean coverage compared to the Roche NimbleGen (43.9×, range 4-145 *vs* 35.6× range 2-107), as already observed. Additionally, both enrichment systems showed no relevant difference comparing FF and FFPE samples within each single region, reporting a similar trend between the two sample types (Agilent: 42.5× ± 7.8 FF *vs* 45.3× ± 9.1 FFPE; Roche: 34.5× ± 9.7 FF *vs* 37.2× ± 8.0 FFPE), with a slight but not-significant increase of coverage in FFPE samples by both technologies (Fig. 5 a, b). Despite the higher mean coverage achieved by Agilent system, its libraries showed a lower uniformity across the amplicons, with a higher number of regions with low read depth (20 amplicons with coverage <20× *vs* 13 of Roche) or very high coverage (10 amplicons with coverage >80× *vs* 2 of Roche) (Fig. 6).

It is worth to mention that both capture systems showed a scarce coverage in *TP53*, one of the most frequently mutated genes in cancer [37, 38], with only 3/8 amplicons with a read depth greater than 20× (Agilent: Chr17:7576996-7577178; Chr17:7578160-7578320; Chr17:7578335-7578503; Roche NimbleGen: Chr17:7577489-7577636; Chr17:7578160-7578320; Chr17:7579330-7579506) (Fig. 6, Additional file 3: Table S7).

**Fig. 7:**
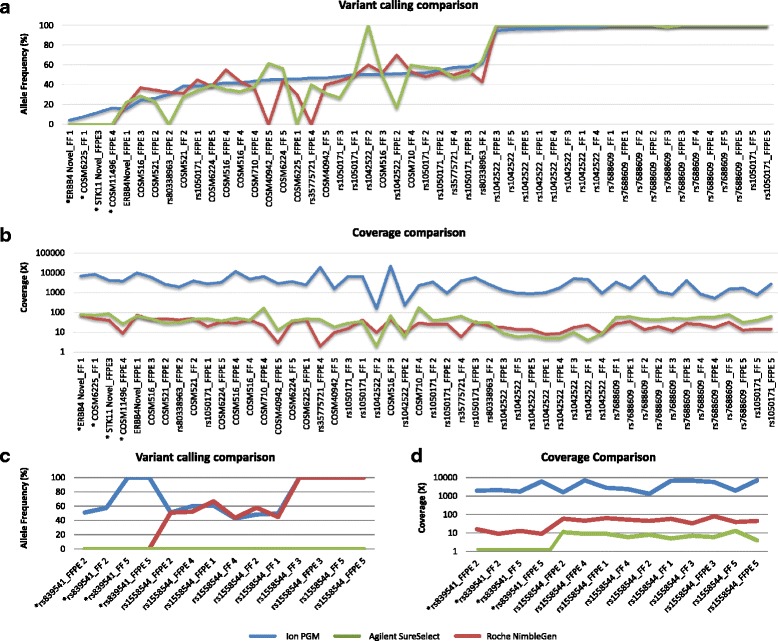
Variant calling comparison between Ion PGM data and both WES systems. Variant calling comparison between Ion PGM data (blue) and both Agilent SureSelect (green) and Roche NimbleGen (red) data in exon regions shows 88 % of concordance (44/50) in both WES capture systems (**a**). Both systems failed to call 4 genetic variants (*) detected by Ion PGM platform at low frequencies (4-16 %). Further 4 variants were missed as follows: 2 by Agilent (COSM6225, rs80338963) and 2 by Roche NimbleGen (COSM40942, rs35775721). Horizontal axis reports the genetic variants (Additional file 3: Table S8a) ordered from lowest to highest frequency (vertical axis) as assessed by Ion PGM platform. Variant coverage displays a quite similar trend between Agilent (green) and Roche NimbleGen (red) libraries, and is far lower than Ion PGM platform (blue) (**b**). Two Roche libraries report a low coverage in the uncalled variants (COSM40942, rs35775721). Vertical axis displays the variant coverage in logarithmic scale. Variant calling comparison between Ion PGM data (blue) and both Agilent (green) and Roche NimbleGen (red) data in non-exon regions shows a poor performance of both WES technologies (**c**). Both WES systems failed to call the rs839541 (*) SNP in *ERBB4* gene, whereas rs1558544 SNP in *EGFR* was missed by all 10 Agilent libraries. Vertical axis reports the frequency of the genetic variants. Variant coverage comparison between Ion PGM data (blue) and both Agilent (green) and Roche NimbleGen (red) data in non-exon (intron/downstream/upstream) regions reports a low coverage in both exome capture kits (**d**); rs839541 SNP was completely uncovered in Agilent libraries. Vertical axis displays coverage values in logarithmic scale

We further assessed the degree of variant calling concordance between WES and the targeted re-sequencing approach. Specifically, the VC plugin on Ion PGM™ data identified a total of 64 genetic variants (50 in exons and 14 in exon-intron junction regions), reporting a 94 % of concordance between FF and FFPE mutational profiles. Two SNVs (NM_000455.4 *(STK11):* c.157G > C, p.Asp53His; NM_000546.5 *(TP53)*: c.476C > A, p.Ala159Asp) were only identified in two FFPE samples (Additional file 3: Table S8) suggesting an intra-tumor heterogeneity as commonly described in lung cancer [39]. Although the average coverage obtained per sample by WES was only 30-40× compared to more than 2000× achieved by the PCR-based kit, both enrichment kits showed a good performance in the exon variant call data, revealed by 88 % of concordance of each kit with Ion data (44 out of 50 exon variants) (Fig. 7 a, b, Additional file 3: Table S8). Additionally, the variant frequency of shared variants was similar between Ion PGM™ and WES data from both kits (Fig. 7a). None of the exome capture systems reported any further variants in the target regions analyzed by Colon and Lung Cancer Panel. We observed that the 4 Ion PGM™ variants missed by the GATK pipeline in both exome capture systems (NM_005235.2 (*ERBB4): c.2784 T > A, p.Glu928Asp*; NM_005228.3 (*EGFR): c.2236_2250del, p.Glu746_Ala750del;* NM_000455.4 (*STK11): c.157G > C, p.Asp53His;* NM_000546.5 (*TP53): c.476C > A, p.Ala159Asp*), were called by Ion pipeline with a low frequency (4.2–16.6 %). However, these variants were successfully confirmed by visual inspection of alignments obtained from both exome kits, with a similar frequency reported by Ion PGM™ (range: 2–10 %). The only exception was *TP53* variant*,* that was missed by Roche NimbleGen system due to an unsuccessful coverage (9× only). Roche failed to call two further variants (NM_001127500.1 (*MET*): c.534C > T, p.(=); NM_000546.5 (*TP53): c.380C > T, p.Ser127Phe*) in two FFPE samples due to unsuccessful coverage (2× and 3×, respectively). Similarly, the Agilent SureSelect system missed a nonsynonymous coding region in *SMAD* (NM_005359.5: c.1081C > A, p.Arg361Ser) and one in-frame deletion in NM_005228.3 (*EGFR*): c.2236_2250del, p.Glu746_Ala750del, due to a variant caller issue; however, the examination of the BAM files by visual inspection confirmed the presence of both alternative alleles. Finally, when we considered the non-exonic variants (intron/downstream/upstream regions), the Agilent SureSelect enrichment kit showed a worse performance, reporting no call among the 14 Ion variants compared to 10/14 detected by the Roche NimbleGen system (Fig. 7 c, d). However, the 14 calls involved only two Single Nucleotide Polymorphism (SNPs), in *EGFR* (NM_005228.3: c.1498 + 22A > T) (10/14) and *ERBB4* (NM_005235.2: c.421 + 58A > G) (4/14), both excluded from the Agilent design although the BAM file visual inspection confirmed the *EGFR* variant. The Roche design did not include *ERBB4* position, thus explaining the failed calls in Roche libraries, despite the *ERBB4* SNP was confirmed by BAM file visual inspection in four positive libraries.

### Coverage of cancer related genes

**Fig. 8:**
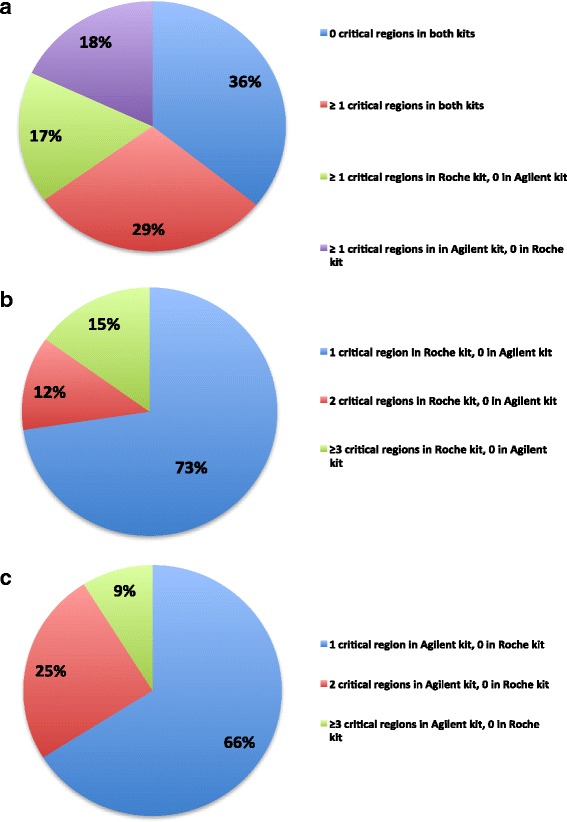
Coverage distribution across all the coding exons of 623 cancer-related genes in both WES platforms. Distribution summary of 623 cancer-related genes according to their coverage performance achieved in the two tested WES systems (**a**). Specifically, 36 % of the genes (red) were completely well covered by both Agilent and Roche kits; 29 % (blue) had at least one ‘critical’ region in both kits; 18 % were completely well covered by Roche NimbleGen kit, but had one or more ‘critical’ region in Agilent SureSelect kit; finally, 17 % of the genes were completely well covered by Agilent SureSelect kit, but had one or more problematic region in Roche NimbleGen kit. Distribution summary of cancer-related genes having one (73 %), two (12 %) or more (15 %) critical regions in NimbleGen Roche kit, but completely well-covered in Agilent SureSelect kit (**b**). Distribution summary of cancer-related genes having one (66 %), two (25 %) or more (9 %) critical regions in Agilent SureSelect kit, but completely well-covered in Roche NimbleGen kit (**c**)

To further assess the WES potential in retrieving clinically relevant genetic variants related to cancer phenotype, we investigated the exon coverage of the most relevant cancer-related genes. Specifically, we selected 623 genes by matching the gene lists of 21 commercialized cancer-specific panels (Additional file 4: Table S9). The coverage distribution across all the coding exons of the selected genes in each library was performed applying the GATK DiagnoseTarget tool, according to the defined criteria. We found that 35.8 % of genes (223/623) showed all coding exons successfully covered by both Agilent and Roche kits (Fig. 8a). Conversely, 29.2 % (182/623) of the genes reported at least one ‘critical’ region in both kits, and 16 out of 182 genes had three or more low coverage regions in both kits. The Roche kit reported further 106/623 genes (17.0 %) with one or more critical regions, in addition to the 182 genes shared with Agilent. Among them, for 4 genes (*MYCN, PBX1*, *RUNX1T1,* and *SEPT9)*, one or more exonic regions were excluded from the Roche target design, although in only one *RUNX1T1* exon a mutation has been reported in the Catalogue of Somatic Mutation in Cancer (COSMIC) database (data not shown) (http://cancer.sanger.ac.uk/cosmic) [40, 41]. Further 75, 11, 13 and 2 genes out of 106 carried one, two, three and four insufficient coverage regions, respectively, in Roche kit (Fig. 8b). Moreover, eight out of 20 exons were flagged as ‘critical’ in one *MST1R* gene. Similarly, 112/623 genes (18.0 %) carried one or more ‘critical’ exonic regions in Agilent libraries only (Fig. 8a).

Agilent design did not include one or more exons containing at least one cancer-linked mutation listed in COSMIC for 4 out of 112 genes *(BRCA1, KMT2C, H3F3A,* and *SSX1*) (data not shown). Furthermore, 73, 26, 4 and 3 genes out of 112 carried, respectively, one, two, three and four low coverage regions in Agilent SureSelect kit; further 2 genes had a higher number of exons with low coverage (*YES1* and *MUC16* carrying 5/11 and 15/84 exonic regions with low read depth, respectively) (Fig. 8c).

We also evaluated the coverage performance of the two kits with respect to the sub-group of the 623 cancer-related genes which were prioritized according to their presence in four databases: Cancer Drivers Database 2014.12 [42], Gene-Drug Knowledge Database *v.*9.0 [43], EXaCT-1 [44] and TARGET *v.*3 [23]. Among the 182 genes with at least one critical region in both WES kits (174 with insufficient coverage and 8 with poor mapping quality), 27 (14.8 %) were reported in at least 3 databases, and 8/27 (*ALK, BRAF, CDH1*, *ERBB2, NOTCH1, PTEN, RB1,* and *TP53*) were also shared in at least 10 re-sequencing panels (Additional file 4: Table S9). Furthermore, 4/27 genes (*BRAF*, *MAP2K4*, *NF1,* and *RB1*) performed worse in Agilent than in Roche kit, i.e. they carry only one ‘critical’ region in Roche libraries compared to four or more problematic regions in Agilent samples (Additional file 4: Table S9). Conversely, Roche kit showed a worse performance in 5/27 genes carrying three (*ALK* and *DNMT3A*), five (*TP53*), six (*JAK3*) and seven (*ERCC2*) critical exons compared to only one critical exon in Agilent libraries (Additional file 4: Table S9). When we focused on the genes with the worst performance in Roche libraries only, we retrieved a list of 25 genes shared in at least 3 databases and among them we found 5 genes having more than three low coverage regions (*BAP1, FLCN, NTRK1, SMARCA4,* and *WT1)* (Additional file 4: Table S9). On the contrary, among the 22 potentially critical genes present in at least 3 databases and incompletely covered in Agilent libraries, only two (*CREBBP* and *NPM1*) reported several regions with low coverage (Additional file 4: Table S9).

Finally, in order to better assess the translational potential of WES data, we also investigated if the presence of low coverage regions within 74 genes previously prioritized (27 in both platforms, 25 in Roche, and 22 in Agilent) could be critical for the occurrence of clinically actionable mutations [43] in those regions. The intersection of data identified a total of 12 mutations linked to therapeutic actions (Additional file 5: Table S10) within 5 low coverage exons in 5 genes (*ALK, JAK3, AR, FGFR2,* and *GNAQ*). The mutations within *ALK*, *AR* and *GNAQ* critical intervals had not uniform coverage across the libraries (the coverage depth at all mutation positions reached values ≤10× in only some libraries). This suggested that the performance of the interval could be library-dependent and not related to a low performance of the bait. In contrast, four mutations (A572V and A573V in *JAK3*; V565I and E566G in *FGFR2*) achieved an extremely low read depth (<8×) in almost all Agilent libraries. Interestingly, in additional four Agilent libraries sequenced at high read depth (mean coverage >100×) in our lab, the variant coverage was similarly unsuitable (data not shown), leading to hypothesize a low performance of the specific capturing baits.

## Discussion

WES applied to FFPE samples in the context of precision medicine and clinical cancer care has been recently described [23, 44] showing the suitability of gDNA extracted from FFPE specimens for library preparation and sequencing [22–28]. However, it is well known that extended formalin fixation could result in highly degraded gDNA [45], possibly unsuitable for downstream applications such as sequencing. Power and limitations of different enrichment platforms should be benchmarked, especially on critical samples such as FFPE specimens, if intended use of WES is in clinical context.

Here we report a comparison study between two in-solution capture platforms, Agilent SureSelect XT *v.5* and Roche NimbleGen *v.3*, analyzing FF and matched FFPE gDNA samples extracted from lung ADC tissues (Additional file 1: Fig. S2). Low amount of degraded gDNA from FFPE samples (300 ng) was not found to be a limiting factor. As WES is still moderately expensive, a QC step on FFPE gDNA should be mandatory, and different methods have been proposed [25, 26]. We evaluated the degradation status of FFPE gDNA with two different methods (PCR-based assay and automated gel-based electrophoresis system) which provided similar information about the FFPE degradation status; FFPE gDNA fragmented up to 70 % could be successfully sequenced. In agreement with our previous study, PCR multiplex assay was a predictor for the success of PCR-based capture re-sequencing [46]; furthermore, a recent application note by Agilent showed similar results in a pilot study on 197 FFPE gDNA, setting a QC cut-off ≥ 3 DIN to proceed to downstream workflow [47]. PCR-based assays have the advantage to outline suitability of FFPE gDNA to amplify specific genomic region sizes according to the library dimension. On the other hand, Agilent 2200 TapeStation system, which reports fragmentation pattern over the whole genome, is optimal in genome wide studies, it is a time-effective alternative to a PCR-based assay and it allows to save gDNA for further downstream applications (~5 ng of gDNA input *vs* 30-100 ng for the multiplex-PCR QC assay).

Analysis of sequencing metrics showed that Agilent SureSelect XT kit achieved a higher ratio of on-target bases, similarly to Shigemizu et al. [21]. We also found a statistically significant increase of duplicated reads in FFPE samples compared to matched FF samples in both kits. This result is possibly a consequence of lower library complexity, which usually occurs when the amount of gDNA is very low or highly degraded, such as FFPE gDNA [24, 36]. Despite the input FFPE gDNA was the same, Roche NimbleGen resulted in even higher sequence duplication rate. Roche NimbleGen libraries reported lower values of mean target coverage; this difference was mainly due to the larger size of its target region (64 Mb *vs* 50 Mb). Both enrichment systems reported high concordance of variant calling between matched FF and FFPE samples while concordance on InDel calls in matched FF-FFPE pairs was lower, probably as result of a low mean coverage [36]. Genotype CR of shared SNVs between the platforms on the common 42 Mb target region was nearly 100 % and it was found high at different coverage thresholds for both platforms, with Roche system revealing slightly decreasing values at higher coverages. We speculate that this behaviour, mirrored by an increasing NRDR, might be related to the intra-pair heterogeneity highlighted when the percentage of target bases covered at least a certain coverage threshold (i.e. ≥ 30×) was taken into account.

DNA artefacts that arise from formalin crosslinking increase false positive calls; treatment with uracil-DNA glycosylase (UDG), included in some gDNA isolation procedures, such as GeneRead DNA FFPE kit (Qiagen), allows the removal of cytosine deamination artefacts, minimizing the risk of false SNP calls [48]*.* We hence investigated the prevalence of known FFPE artefacts (C > T and G > A substitutions) in FFPE samples treated with UDG. In general, CR and NRDR values computed for C > T and G > A substitutions were found in line with other transition rates. FFPE artefacts are estimated to happen at a rate ~10 %, a level similar to sub-clonal mutations in heterogeneous cancer samples, and can be highlighted with high coverage data [48]. In line with this finding, we found that spurious calls due to cytosine deamination can only be identified at the highest coverages, although absolute rates remain low when UDG treatment is applied. Despite the encouraging results obtained with gDNA extracted from FFPE samples, we cannot exclude that the library preparation starting from lower quality input gDNA samples might be more challenging [24].

By hypothesizing the use of WES data in a clinical context, we also evaluated the performance in variant detection of both the enrichment systems over 22 lung cancer-related genes (90 amplicons) included in the AmpliSeq Colon and Lung Cancer Panel *v.*1, using sequencing data obtained from Ion Torrent PGM™ platform as positive control. We observed that Agilent libraries reported lower read depth uniformity across the 90 amplicons, despite the higher mean coverage over the whole exome target region. The same response has been also reported as a platform bias in previous versions of the kit [15, 49]. Our comparison analysis displayed that nearly 90 % of variants detected by the Ion Torrent platform were correctly called with a similar frequency in both platforms, without false positives, irrespective of the type of input gDNA. This good concordance was achieved despite the uneven coverages on the two sequencing systems (~30×-40× on HiSeq *vs* ~2000× on Ion Torrent platform). Both enrichment systems showed their potential of retrieving clinically actionable single nucleotide substitutions, e.g. COSM6224 linked to the activity of EGFR inhibitors [50, 51], except for those spanning exon-intron junctions, due to missing probes in their target designs. We finally challenged the two enrichment platforms in the characterization of 623 cancer-related genes selected from 21 commercial gene panels. While both kit designs covered almost all the exonic regions of those genes, with nearly half of them efficiently captured, we were able to describe few genes marked by one or more low coverage exons that could be critical for therapeutic targeting. Overall, the ability of the two kits to efficiently cover cancer-related genes in both FF and FFPE samples is satisfactory and comparable between the two systems. We speculate that WES approach, which allows the analysis of all cancer genes under investigation, could be an efficient alternative option compared to target re-sequencing panels with the major advantage it allows to describe the mutational landscapes linked to tumor progression, novel drug resistance-associated mutations and even assist therapeutic choice due to the rapid rate of novel targeted therapies development [44].

## Conclusions

Our data substantiate the feasibility of generating high-quality libraries and sequencing data from relatively low input of highly fragmented FFPE gDNA, without significant differences between the two tested platforms.

We also demonstrate that each WES platform is able to correctly detect most of the SNVs detected by a PCR capture re-sequencing, without introducing false negative results. Furthermore, both WES capture systems efficiently cover almost all exons of the most cancer-relevant genes.

Therefore, our study demonstrates that FFPE samples may replace the frozen tissues in a WES workflow, although a QC step of FFPE degradation status should be integrated as a decision criterion to proceed for the sequencing.

Researchers should keep in mind that the WES designs continuously evolve and both technologies recently released new versions namely Human All Exon *v.*6 (Agilent Technologies) and SeqCap EZ MedExome Kit (Roche NimbleGen) that have been optimized in design with an improvement of the disease-linked variant detection.

In conclusion, our analysis suggests that the WES approach could be extended to a translational research context as well as to the clinic (e.g. to study rare malignancies), where the simultaneous analysis of the whole coding region may help in the detection of cancer-linked variants.

## Additional files

Additional file 1: Figure S1 and Figure S2.
**Figure S1.** DNA quality control. TapeStation profiles of gDNA isolated from FF and matching FFPE block tumor tissues from 5 lung ADC patients. In each profile, the DIN, indicative of gDNA degradation status, is also displayed (numerical assessment ranges from 10 for undamaged gDNA, to 1 for highly fragmented gDNA) **(a)**. The Table reports the gDNA concentration (ng/ul) assessed by NanoDrop, Qubit, and TapeStation, and purity (260/280 and 260/230) (**b**). Additionally, AYR and DIN parameters, indicative of FFPE gDNA fragmentation status, evaluated by a multiple PCR assay and TapeStation respectively, are reported. Image of agarose gel 1 % shows the gDNA smears indicative of the different degradation status of FF and FFPE gDNAs **(c)**. **Figure S2.** The workflow illustrates samples processing and WES data analysis for both exome enrichment platforms. (PDF 187 kb) Additional file 2: Table S1, Table S2, Table S3, Table S4, Table S5, and Table S6. 
**Table S1.** Sequencing metrics for libraries prepared with both Agilent SureSelect XT *v.*5 and Roche NimbleGen *v.*3.0  kits starting from five matched FF and FFPE tumor samples. **Table S2.** Variant detection comparison between matched FF-FFPE pairs. For each matched FF-FFPE pair, the number and the percentage of both SNVs and InDels common to both sample types, and unique to either FF or FFPE sample are reported. **Table S3.** Genotype CR and NRDR between matched FF-FFPE pairs at increasing coverage thresholds. For each matched FF-FFPE pair, the genotype CR was computed as the ratio between the sum of concordant genotypes and the sum of all genotypes called at genomic positions covered at least a certain coverage threshold (from 1 to 50×) in both samples (a). For each matched FF-FFPE pair, the NRDR was computed as the ratio between the sum of non-concordant genotypes and the sum of all non-reference genotypes called at genomic positions covered at least a certain coverage threshold (from 1 to 50×) in both samples (b). **Table S4.** Genotype CR and NRDR between matched FF-FFPE pairs computed for each transition type at increasing coverage thresholds. For each matched FF-FFPE pair, the genotype CR for each transition type was computed as the ratio between the sum of concordant genotypes and the sum of all genotypes called at genomic positions covered at least a certain coverage threshold (from 1 to 50×) in both samples; p-values for two-tail *t*-test for each comparison between two transition types are reported at the bottom of the table (a). For each matched FF-FFPE pair, the NRDR for each transition type was computed as the ratio between the sum of non-concordant genotypes and the sum of all non-reference genotypes called at genomic positions covered at least a certain coverage threshold (from 1 to 50×) in both samples; p-values for two-tail *t*-test for each comparison between two transition types are reported at the bottom of the table (b). **Table S5.** Variant detection comparison between exome libraries prepared with both Agilent SureSelect and Roche NimbleGen kit. The table reports the total number and the percentage of SNVs and InDels common to both library prep types for each sample, and unique to either Agilent SureSelect and Roche NimbleGen kit. The comparison was performed considering both the whole kit-specific target region and the 42 Mb of common target region. **Table S6.** Genotype CR and NRDR rates within the shared 42 Mb target region between Agilent SureSelect and Roche NimbleGen at increasing coverage thresholds. For each sample, the genotype CR was computed as the ratio between the sum of concordant genotypes and the sum of all genotypes called at genomic positions covered at least a certain coverage threshold (from 1 to 50×) in both Agilent SureSelect and Roche NimbleGen libraries (a). For each sample, the NRDR was computed as the ratio between the sum of non-concordant genotypes and the sum of all non-reference genotypes called at genomic positions covered at least a certain coverage threshold (from 1 to 50×) in both in both Agilent SureSelect and Roche NimbleGen libraries (b). (XLSX 54 kb) Additional file 3: Table S7 and Table S8. 
**Table S7.** Mean coverage achieved by Agilent SureSelect and Roche NimbleGen libraries within 90 PCR-capture amplicons. Mean coverage ± SD within 90 regions amplified by AmpliSeq Colon and Lung Cancer Panel *v.*1 (Thermo Fisher Scientific) from ‘FF’, ‘FFPE’ and ‘FF plus FFPE’ samples achieved by Agilent SureSelect and Roche NimbleGen libraries respectively. In each column, the mean coverage values are reported for each amplicon, and the heat map was created using two-color scale (lowest value is represented by dark blue and highest value by dark red). **Table S8.** Variant calling comparison between the two WES systems (Agilent SureSelect and Roche NimbleGen) and the AmpliSeq Colon and Lung Cancer Panel. List of FFPE and matched FF samples genetic variants called by VC *v.*4.2 plugin on Ion PGM^TM^ data and GATK pipeline in both exome capture systems. All variants are annotated with gene ID, locus, reference sequence, variant allele according to the hg19 Reference Genome. The red bars show the variant allele frequency (%) detected by VC on Ion pipeline and GATK on both Agilent SureSelect and Roche NimbleGen WES (0* means variant not called but found by IGV visual inspection of BAM files). All variants are annotated for COSMIC or dbSNP (rs number) together with the codons involved and the amino acid change (AA). The 'Effect' column reports if the variant is in a coding region, discerning between nonsynonymous, synonymous and non-sense, or in an intron, downstream the gene or in a splicing region. The last four columns of the table reports the Minor Allele Frequency (MAF) reported in the 1000 Genomes Project, the prediction effect on the protein based on SIFT and Polyphen algorithms and the conservation score namely GERP. For SIFT prediction, the higher the number, the lower is the effect, whereas for Polyphen prediction is the opposite. Thus, a higher score for GERP indicates a higher conservation of the gene across 34 mammalian species. *Abbreviation:* - not available data. (XLSX 44 kb) Additional file 4: Table S9. Coverage distribution across all the coding exons of the 623 cancer related genes in each library. For each gene, the table reports the number of coding RefSeq exons downloaded from UCSC, their presence within 21 commercial re-sequencing cancer panels and further four cancer genes databases. The coverage distribution across all coding exons was performed using the GATK DiagnoseTarget tool. For each WES capture platform we reported: the number of ‘critical’ exons (average depth of coverage < 10× for at least 20 % of the length of the interval and with insufficient median depth across all FF and FFPE libraries), the number of exon regions missed by the kit target design file, and the % of passed exons (average depth of coverage ≥ 10× for at least 20 % of the length of the interval). (XLSX 120 kb) Additional file 5: Table S10. Clinically actionable mutations within low coverage exons of database-prioritized cancer related genes. For each selected genomic interval the table reports the mean coverage and SD values in both Agilent SureSelect and Roche NimbleGen libraries, and the list of clinically actionable mutations belonging to that interval, retrieved from Gene-Drug Knowledge Database v9.0 [43]. (XLSX 13 kb)

## Abbreviations

ADC: Adenocarcinoma
AYR: Average yield ratio
BAM: Binary Alignment/Map
BWA-MEM: Burrows-wheeler aligner maximal exact match
COSMIC: Catalogue of somatic mutation in cancer
CR: Concordance rate
DIN: DNA integrity number
FF: Fresh-frozen
FFPE: Formalin-fixed paraffin-embedded
*GAPDH*: Glyceraldehyde-3-phosphate dehydrogenase
GATK: Genome analysis toolkit
gDNA: Genomic DNA
IGV: Integrative genomics viewer
InDel: Insertion/Deletion
NGS: Next generation sequencing
NRDR: Non-reference discordance rate
PCR: Polymerase chain reaction
PGM: Personal genome machine
QC: Quality control
SNP: Single nucleotide polymorphism
SNV: Single nucleotide variant
UDG: Uracil-DNA glycosylase
VC: Variant caller
WES: Whole-exome sequencing
