# Supplementary material for: Performance comparison of two commercial human whole-exome capture systems on formalin-fixed paraffin-embedded lung adenocarcinoma samples
Source: BMC Cancer. 2016 Aug 30;16(1):692. doi: 10.1186/s12885-016-2720-4 (PMC5004269; doi:10.1186/s12885-016-2720-4)
Supplement: Additional file 1: Figure S1 and Figure S2. — Figure S1. DNA quality control. TapeStation profiles of gDNA isolated from FF and matching FFPE block tumor tissues from 5 lung ADC patients. In each profile, the DIN, indicative of gDNA degradation status, is also displayed (numerical assessment ranges from 10 for undamaged gDNA, to 1 for highly fragmented gDNA) (a). The Table reports the gDNA concentration (ng/ul) assessed by NanoDrop, Qubit, and TapeStation, and purity (260/280 and 260/230) (b). Additionally, AYR and DIN parameters, indicative of FFPE gDNA fragmentation status, evaluated by a multiple PCR assay and TapeStation respectively, are reported. Image of agarose gel 1 % shows the gDNA smears indicative of the different degradation status of FF and FFPE gDNAs (c). Figure S2. The workflow illustrates samples processing and WES data analysis for both exome enrichment platforms. (PDF 187 kb) [file 12885_2016_2720_MOESM1_ESM.pdf]

Fig. S1

a

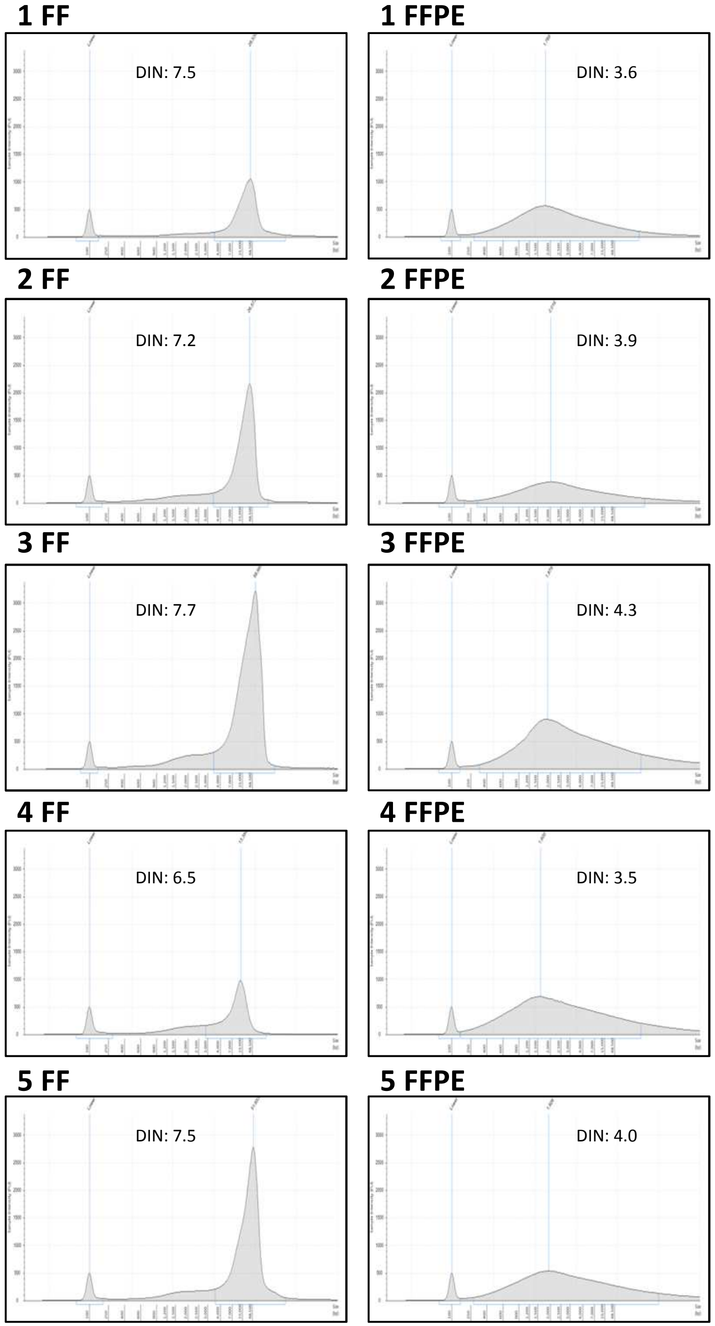

b

Patient ID	Tumor Type	NanoDrop [ng/ul]	260/280	260/230	QuBit [ng/ul]	AYR	TapeStation [ng/ul]	DIN	Range Size (bp)	Peak Size (bp)
1	FF	87.6	1.9	1.9	41.7	n.d.	64.6	7.5	3491- >60000	28534
	FFPE	388.5	1.8	2.2	41.7	0.53	133.0	3.6	273- >60000	1789
2	FF	422.3	1.9	2.1	41.0	n.d.	111.0	7.2	3410- >60000	26573
	FFPE	209.2	1.9	2.2	53.0	0.56	88.3	3.9	298- >60000	2016
3	FF	582.5	1.8	2.2	46.0	n.d.	219.0	7.7	3445- >60000	58987
	FFPE	385.0	1.8	2.2	67.6	0.68	217.0	4.3	321- >60000	1879
4	FF	68.5	1.8	2.3	27.8	n.d.	44.2	6.5	2880- >60000	13394
	FFPE	368.8	1.8	2.2	45.1	0.53	185.0	3.5	154- >60000	1600
5	FF	237.9	1.8	2.2	59.0	n.d.	133.0	7.5	3563- >60000	51653
	FFPE	460.1	1.8	2.2	55.0	0.61	149.0	4.0	270- >60000	1926

c

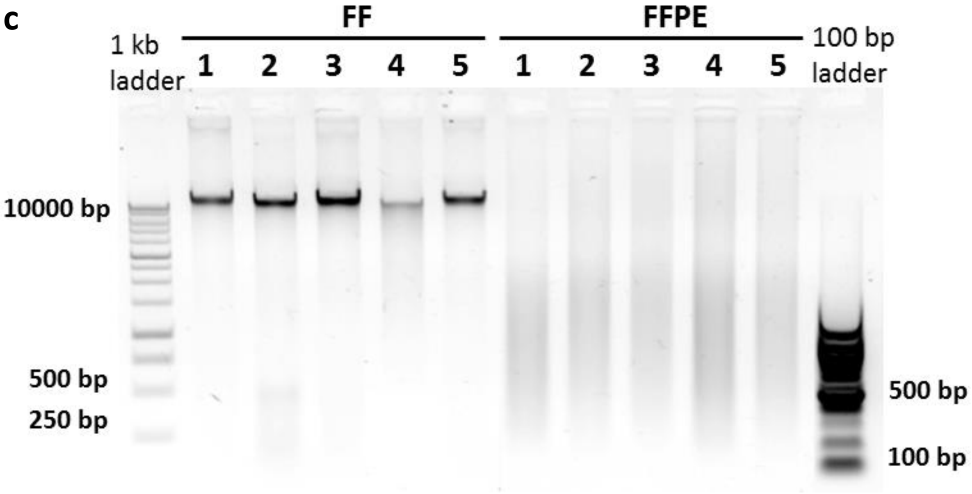

Fig. S2

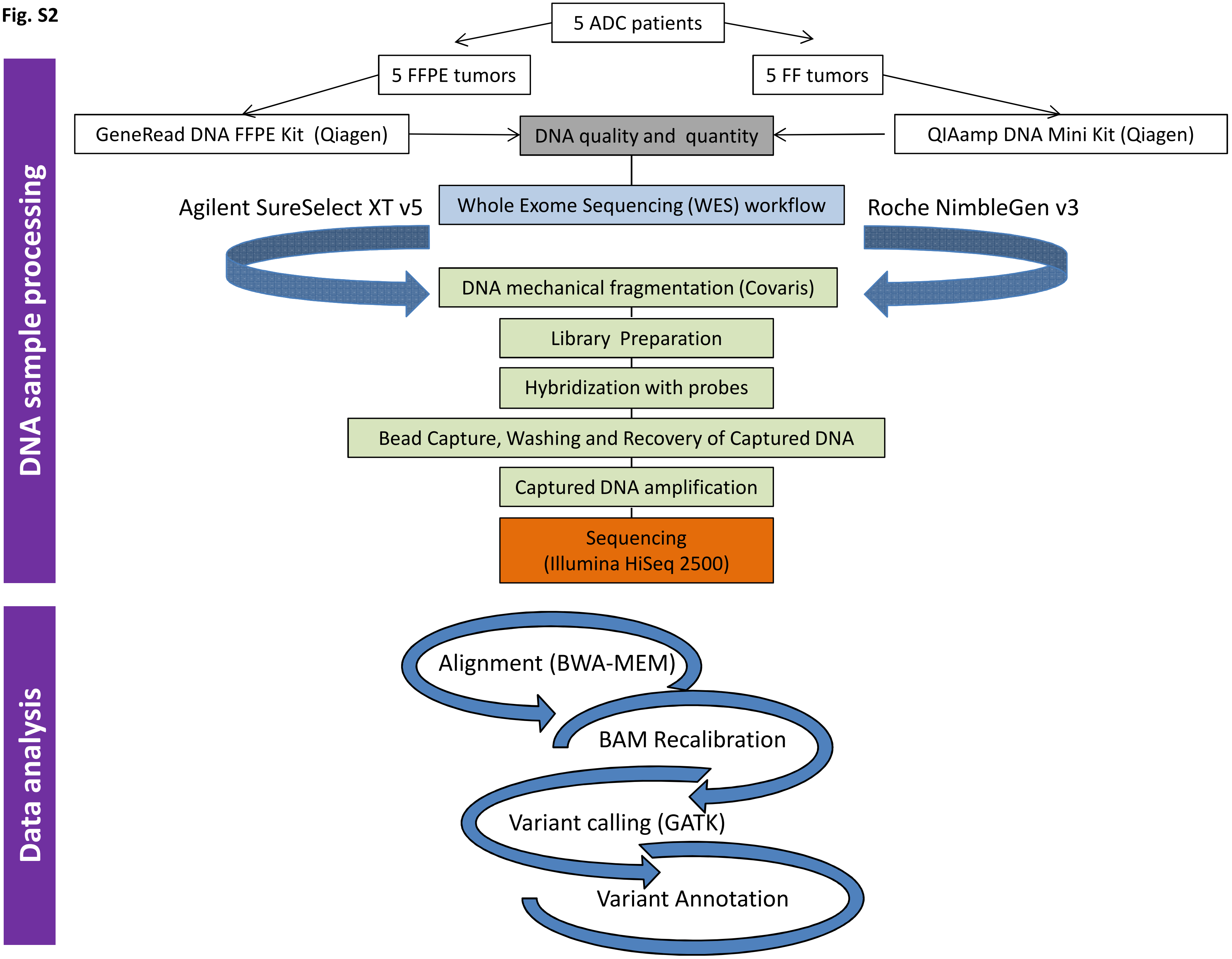
DNA sample processing
5 ADC patients
5 FFPE tumors
5 FF tumors
GeneRead DNA FFPE Kit (Qiagen)
DNA quality and quantity
QIAamp DNA Mini Kit (Qiagen)
Agilent SureSelect XT v5
Whole Exome Sequencing (WES) workflow
Roche NimbleGen v3
DNA mechanical fragmentation (Covaris)
Library Preparation
Hybridization with probes
Bead Capture, Washing and Recovery of Captured DNA
Captured DNA amplification
Sequencing
(Illumina HiSeq 2500)
Data analysis
Alignment (BWA-MEM)
BAM Recalibration
Variant calling (GATK)
Variant Annotation
